# O.1.3-8 How reliable, valid and readable are brief physical activity questionnaires? Results of a systematic review

**DOI:** 10.1093/eurpub/ckad133.105

**Published:** 2023-09-11

**Authors:** Antonina Tcymbal, Karim Abu-Omar, Sven Messing, Peter Gelius, Ivo Rakovac

**Affiliations:** Friedrich-Alexander Universität Erlangen-Nürnberg; Friedrich-Alexander Universität Erlangen-Nürnberg; Friedrich-Alexander Universität Erlangen-Nürnberg; Friedrich-Alexander Universität Erlangen-Nürnberg; WHO European Office for the Prevention and Control of Noncommunicable Diseases; antonina.tcymbal@fau.de

## Abstract

**Purpose:**

Accurate and fast measurement of physical activity is important for surveillance. However, it is currently not clear which of the brief physical activity questionnaires (PAQ) is the most reliable, valid and easy to use. The aim of this systematic review was to identify existing brief PAQs, as well as to describe and compare their measurement properties and level of readability.

**Methods:**

We performed a systematic review based on the PRISMA statement. Literature searches were conducted in six scientific databases in March 2022. Articles were included if they evaluated validity and/or reliability of brief (i.e. with a maximum of three questions) physical activity or exercise questionnaires intended for healthy adults. Due to the heterogeneity of studies, data were summarized narratively. The level of readability was calculated according to the Flesch-Kincaid formula.

**Results:**

Overall, 34 articles published in English or Spanish were included, assessing 31 unique brief PAQs. Overall, studies showed moderate to good reliability levels of the PAQs. The majority of results showed weak validity of brief PAQs against objective measurements and weak to moderate validity against other PAQs. Most of the assessed PAQs met the criterion of “short” and can be read by respondents themselves or aloud by an interviewer in less than one minute. However, only 17 questionnaires have a readability level that can be easily understood by the majority of the population.

**Conclusions:**

This review identified numerous different brief PAQs, but most of them were evaluated with only a single study. Ways of assessing measurement properties varied widely between studies, limiting comparability between PAQs and making it difficult to suggest one tool as the most suitable. Questionnaires use different concepts of measuring PA so that the choice of tool will be based not only on measurement properties but also on assessment aims. Future developments or adaptations of PAQs should consider readability levels as an important factor.

**Support/Funding Source:**

no outside funding.

